# Genetic Evidence for a Potentially New Pathogenic *Leptospira* sp. Circulating in Bats from Brazilian Amazon

**DOI:** 10.1155/2023/9677047

**Published:** 2023-09-19

**Authors:** Maria Isabel Nogueira Di Azevedo, Rair de Souza Verde, Camila Ezepha, Filipe Anibal Carvalho-Costa, Paulo Sérgio D'Andrea, Luciana dos Santos Medeiros, Walter Lilenbaum

**Affiliations:** ^1^Laboratory of Veterinary Bacteriology, Biomedical Institute, Federal Fluminense University, Niteroi, Brazil; ^2^Postgraduate Program in Health and Sustainable Animal Production in the Amazon, Federal University of Acre, Acre, Brazil; ^3^Laboratory of Epidemiology and Molecular Systematics, Oswaldo Cruz Institute, Rio de Janeiro, Brazil; ^4^Laboratory of Biology and Parasitology of Wild Reservoir Mammal, Oswaldo Cruz Institute, Oswaldo Cruz Foundation, Rio de Janeiro, Brazil; ^5^Department of Veterinary Collective Health and Public Health, Federal Fluminense University, Niteroi, Brazil

## Abstract

Leptospirosis is a neglected zoonosis frequently reported worldwide, caused by a spirochete of the genus *Leptospira*. It is capable of infecting domestic animals, free-living animals, and humans. Among wild animals, the role of bats in the epidemiology of leptospirosis has been investigated but is far from being elucidated. The Amazonian biome has the ideal conditions for maintaining and disseminating leptospires and, despite efforts that have been made to better understand leptospires' occurrence in wild animals in the region, few studies aimed to explore and genetically characterize leptospires in bats. Based on this, the aim of the present study is to deeper investigate and genetically characterize leptospires detected in bats from the southwest Amazon. Animals were trapped by mist netting at five sites in the state of Acre, Brazil. Kidney samples were obtained and stored for molecular analysis. Polymerase chain reaction (PCR) was conducted first based on the *Lip*L32 gene, and positive samples were submitted to *rrs* and *sec*Y-PCR and sequencing. Sequences were then submitted to phylogenetic analyses through multiple bioinformatic tools. The *rrs* sequences from the present study formed one single haplotype, different from any other previously deposited, grouped in a highly supported cluster with sequences from bats from Madagascar and China. The initial *sec*Y screening revealed no identity with previously deposited sequences. The phylogenetic trees revealed the sequences from the present study in an isolated branch, clearly separated from all previously known pathogenic *Leptospira* spp., suggesting the existence of a potentially undescribed species. The haplotype network including only leptospires from the Amazon region confirmed two new haplotypes from the same taxon unity, isolated from the others, with a probable origin of the species from *L. noguchii*. The characterization of this potentially new species in bats reinforces the complexity of the transmission dynamics of leptospires, including wild, periurban, and urban environments, emphasizing the need for an integrative look at leptospirosis vigilance within the context of One Health.

## 1. Introduction

Leptospirosis is a neglected zoonosis frequently reported worldwide, caused by a spirochete of the genus *Leptospira* sp. [1]. Several species are recognized as pathogenic agents, including over 250 serovars [2]. It is endemic in tropical and subtropical regions of the world and capable of infecting domestic animals, free-living animals, and humans [3]. The bacterium is transmitted through water, soil, or mud contaminated by the urine of infected animals and circulates in urban, rural, and wild environments, with a wide variety of animal species acting as carriers/hosts [4].

Among wild mammals, although rodents are the most important and studied reservoirs [5], bats have been proven to carry *Leptospira* sp. [6], and their role in the epidemiology of leptospirosis has been increasingly investigated over the last two decades [7]. These flying mammals are distributed worldwide and present essential and complex ecological roles, with different feeding habits as, insectivorous, nectarivores, frugivorous, carnivorous, and hematophagous [8].

The Amazonian biome has the ideal conditions for maintaining and disseminating leptospires, such as a warm and humid climate, the presence of great mammal biodiversity, and a variety of potential host species [9]. Many efforts have been made to better explore leptospires' occurrence in wild animals in the region [10–14]. Regarding bats, few studies aimed to explore and genetically characterize leptospires in this region, although a high prevalence has been reported [15, 16].

Recently, our group performed a field study involving the investigation of pathogens in bats from the Brazilian Amazon biome (data not published). Among the many results found, one was particularly intriguing, regarding the possible identification of a new species of *Leptospira* (*Leptospira* n. sp.). Based on this, the aim of the present study is to deeper investigate and genetically characterize these leptospires detected in bats from the southwest Amazon.

## 2. Material and Methods

This study is part of a long-term ecological and parasitological wild mammal project in the Amazon Biome, and has captured 129 bats specimens. All capture, handling, and euthanasia procedures were approved by the Ethics Committee on Animal Use of the Federal University of Acre (CEUA/UFAC under no 28/2019) and under the capture license granted by the competent environmental agency (SISBio under no 71451). The animals were euthanized for other parasitological and taxonomic studies and not only for the purposes of the present work.

### 2.1. Study Area

**Figure 1:**
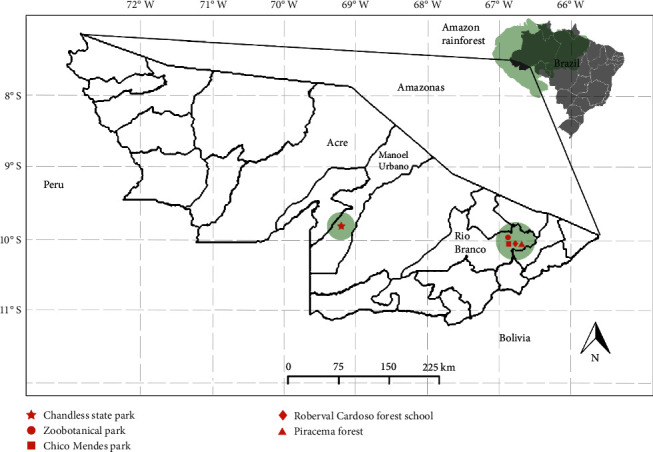
Study areas where bats were captured in the State of Acre, Brazil. The symbols represent locations, which are shown in figure.

This study was conducted during the spring of 2019 and 2021 in four Amazon Forest conservation units, in the state of Acre, Brazil: (1) Chandless State Park (9° 91′ 84″S/70° 15′ 96″W), at municipality of Manoel Urbano, that is characterized as a continuous forest with an area of 695.303 hectares, surrounded by a mosaic of preserved areas; (2) Zoobotanical Park (9° 95′ 70″S/67° 87′ 45″W) at UFAC, of 150 hectares; (3) Chico Mendes Park (10°03′ 71″S/67° 79′ 68″W), of 50 hectares; and (4) the Roberval Cardoso Forest School (10° 05′ 05″S/67°59′15.14″W), and Piracema Forest, of 153 hectares (10° 01′ 42″S/67°55′23.80″W) being the last two at the municipality of Rio Branco, and both characterized as very altered urban forest fragments (Figure 1). The east of the Acre state is dominated by induced pastures for cattle production, where forest cover (primary forest and secondary vegetation) is limited to numerous small patches. In contrast, Chandless State Park, which served as the control area for this study, is characterized by dense shoulder length forests dominated by palm trees.

### 2.2. Capture, Taxonomic Identification and Bat Sample Collection

For the sampling, eight mist nets (12 × 3 m, mesh 19 mm, Ecotone®) were installed for two consecutive nights in two plots in each area at ground level. The captures began at sunset and ended six hours after the installation of the nets, with inspections every 20 min. The captured bats were placed in containment cotton bags for weighing, body measuring, and preliminary taxonomic identification at the genus level. Bats were identified in the field according to the following keys: Gardner [17], Díaz et al. [18], and López-Baucells [19].

The animals were taken to a field laboratory for euthanasia (9 : 1, 10% ketamine hydrochloride and 2% acepromazine) and biological sample collection. The specimens were identified at the species level by external morphological characters and confirmed by molecular analysis (*Cyt*B gene) and DNA sequence comparisons, and further deposited as voucher specimens in the Laboratory of Biology and Parasitology of Reservoirs of Wild Mammals Collection (Oswaldo Cruz Foundation/Rio de Janeiro, Brazil).

### 2.3. DNA Extraction and Molecular Detection of Pathogenic *Leptospira*

All molecular and genetic analyses were based on the previous protocols of our group, as described in [20]. Kidney samples were obtained during the necropsy and stored in sterile 2.0 mL microtubes at −20°C, which were destined for molecular analysis. DNA extraction (kidney samples) was performed using the DNeasy® Blood & Tissue Kit (Qiagen, California, USA), according to the manufacturer's instructions. Specific primers of the *Lip*L32 gene, reported to be present in pathogenic leptospires were used for the reactions [21] (*Supplementary 1*), performed as previously described by Hamond et al. [22]. For each test, ultrapure water was used as a negative control in all reactions, while 10 fg of DNA extracted from *Leptospira interrogans* serovar Copenhageni (Fiocruz L1-130) was used as a positive control. The polymerase chain reaction (PCR) products were analyzed by electrophoresis in 1.5%–2% agarose gel after gel red staining and then visualized under ultraviolet (UV) light.

### 2.4. DNA Sequencing and Phylogenetic Analysis

Samples were submitted to a nested PCR targeting the 16 S rRNA gene (*rrs*) [23] and *sec*Y gene [24]. Detailed information about primers used for *Leptospira* sp. identification in the present study is shown in *Supplementary 1*. Amplicons were purified with the Wizard® SV Gel Kit and PCR Clean-Up System (Promega, USA), according to the manufacturer's instructions and intended for sequencing. Sequencing reactions were performed using the Big Dye Terminator v. 3.1 Cycle Sequencing Kit (Applied Biosystems, USA) on a 3100 automatic DNA sequencer according to the manufacturer's instructions. Regarding sequencing analysis, Pairwise/Blast/NCBI software, SeqMan v. 7.0, ClustalW v. 1.35, and BioEdit v. 7.0.1 were used to edit and analyze the sequences.

Reference sequences of pathogenic *Leptospira* sp. from bats identified worldwide and from different hosts from the Amazon region were obtained from GenBank for *rrs* and *sec*Y analyses, respectively. Unpublished and too short sequences (<400 bp) were excluded to avoid biases in the analyzes. Neighbor-joining (NJ) and maximum-likelihood (ML) trees were constructed using the Tamura–Nei model (TN92) in MEGA X software [25], as it was determined to be the best-fitting model of DNA substitution using the Bayesian information criterion. Genetic distances were calculated using the TN92 model on MEGA X. Nucleotide sequences were translated to amino acid sequences on BioEdit v. 7.0.1 software.

A haplotype network based on *sec*Y sequences from *Leptospira* spp. from the Amazon region was constructed through the population genetics software PopART [26], using the media-joining inference method [27], in order to better visualize the number of single nucleotide polymorphisms (SNPs) and to evaluate haplotypes distribution and evolutionary origins.

## 3. Results

### 3.1. Detection of Pathogenic *Leptospira* sp. in Bats

Pathogenic *Leptospira* sp. was detected through positive *Lip*L32-PCR in bats' kidneys from the species *Carollia perspicillata* (*n* = 1), *Myotis riparius* (*n* = 1), *Choeroniscus minor* (*n* = 1), *Artibeus planirostris* (*n* = 2), *Uroderma bilobatum* (*n* = 1), *Gardnerycteris crenulatum* (*n* = 1), and *Desmodus rotundus* (*n* = 1). Details about positive hosts are summarized in Table 1.

### 3.2. Phylogenetic Analysis

*Lip*L32-PCR positive samples were submitted to *rrs* and *sec*Y-nested PCR. Amplicons with the expected size were produced (600 and 409 bp, respectively), purified, and submitted to DNA sequencing. In all samples, it was possible to obtain high-quality *sec*Y gene sequences, while for the *rrs* gene, it was possible to obtain only three sequences. Sequences were deposited on GenBank under accession numbers OQ793707 and OQ793714. Initially, Pairwise/Blast/NCBI comparisons of the *rrs* sequences from the present study with the GenBank dataset revealed 98% of identity with *L. noguchii*, *L. kirshnerii*, and *L. interrogans*, while no high identity (>95%) was observed with any previously deposited sequences based on *sec*Y sequences.

**Figure 2:**
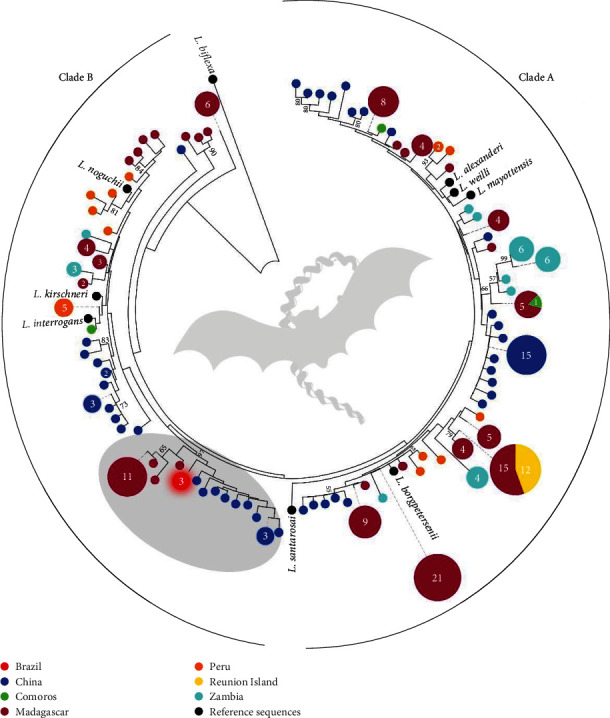
Neighbor-joining (NJ) phylogenetic tree inferred from *rrs* gene sequences (*n* = 249, 440 bp), including pathogenic *Leptospira* sp. previously identified in bats from different geographical localizations in the world (countries are represented by colors, as shown in the legend). The circles represent haplotypes and the numbers inside represent the “*n*” of sequences. The smaller circles without numbers indicate haplotypes with only one sequence. Sequences from the present study are represented by a red circle with a glow. The gray circle indicates the clade formed by sequences from the present study and those previously deposited on GenBank. Numbers at nodes are bootstrap values greater than 50%. *Leptospira biflexa* is the outgroup taxa. Detailed information about sequences included in the analysis is shown in *Supplementary 2*.

The phylogenetic tree based on bat *Leptospira* sp. *rrs* sequences from different geographical origins revealed two main clades, the first including *L. alexanderi*, *L. weilli*, *L. mayottensis*, *L. borgpetersenii*, and *L. santarosai* and the second including *L. noguchii*, *L. kirshnerii* and *L. interrogans* (Figure 2). Sequences from the present study formed a single haplotype, different from any other previously deposited (red circle with glow in Figure 2), grouped in a high supported cluster with sequences from bats from Madagascar and China identified only as *Leptospira* sp. (gray circle in Figure 2). Details about *Leptospira* sp. *rrs* sequences from bats used in this analysis are shown in *Supplementary 2*.

Regarding *sec*Y genetic distances, sequences from the present study presented the lowest distance with *L. noguchii* (TN92 = 0.12 ± 0.02), reflecting only 88% of identity. For the remaining pathogenic species, genetic distances vary from 0.13 to 0.25 (87%–75% of identity) (Table 2). Concerning only the sequences from the present study, intraspecific genetic distance was 0.01, reflecting a high identity between them (99%) (Table 2). The intraspecific genetic distance of *L. noguchii*, the genetically closest species, increased from 0.02 to 0.05 when sequences from the present study were included, a value higher than all intraspecific distances of all species (see values in italics in Table 2).

**Figure 3:**
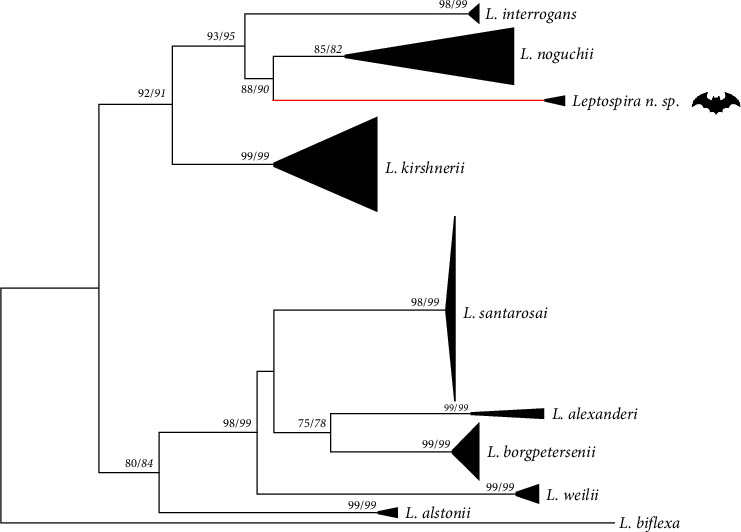
Maximum-likelihood (ML) phylogenetic tree inferred from *sec*Y gene sequences, including the main pathogenic *Leptospira* species and sequences from this study (represented by the red branch). Numbers at nodes are bootstrap values greater than 50%. Regular font numbers correspond to ML analysis and italic numbers to NJ analysis. The size of the triangle reflects intraspecific diversity. *Leptospira biflexa* is the outgroup taxa.

The phylogenetic trees revealed the sequences from the present study in an isolated, highly supported branch (NJ = 99%, ML = 99%), basal to *L. noguchii* species cluster, clearly separated from all previously known pathogenic *Leptospira* sp., suggesting a potential existence of an undescribed species (Figure 3). Moreover, it is possible to observe the cluster from the present study included in a major clade that includes, beyond *L. noguchii*, *L. interrogans*, and *L. kirshnerii* (Figure 3). The *sec*Y amino acid alignment using *L. interrogans* strain Fiocruz L1-130 (AE016823) sequence as a reference and including all *L. noguchii* sequences, showed three genetic signatures, that is, polymorphisms shared only by strains from *Leptospira* n. sp.: T3M, I4V, and V47I. Additionally, the haplotype including the strains R21960, R22023, and R21980 showed an additional signature: L96F. Importantly, the amino acid composition was distinct between *L. noguchii* and *Leptospira* n. sp. from the present study (*Supplementary 3*).

**Figure 4:**
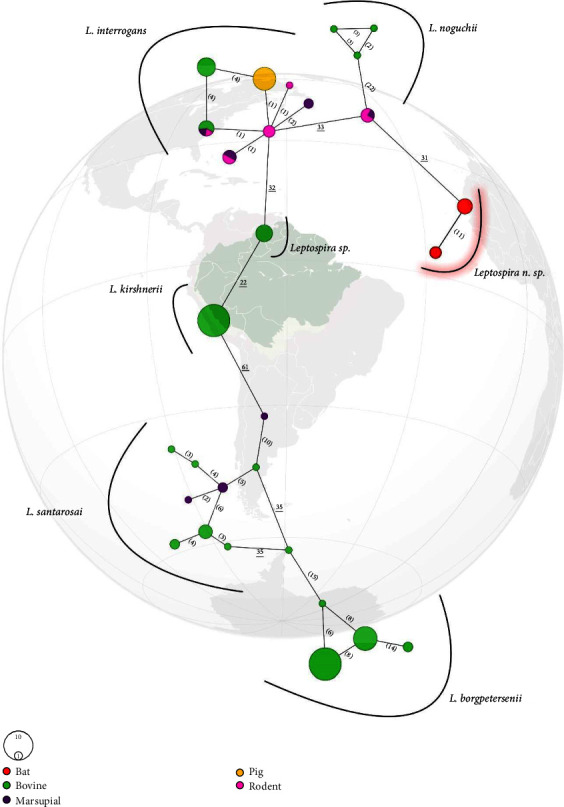
Haplotype network based on *Leptospira* sp. *sec*Y locus identified in hosts from Amazon region (*n* = 125). The colors indicate hosts and the area of the circle is proportional to number of sequences. The number of single nucleotide polymorphisms are shown between parenthesis (intraspecies) and underlined (interspecies). Haplotype from the present study is highlighted in red.

The haplotype network including only *Leptospira* sp. from the Amazon region confirmed two new haplotypes including sequences from bats of the present study, isolated from the others, with a probable origin from *L. noguchii* (Figure 4). It is important to note that the number of SNPs separating the present *Leptospira* n. sp. from *L. noguchii* (*n* = 31) is much bigger than any intraspecific SNP and in accordance with SNPs between species (Figure 4). Moreover, the number of SNPs between the two haplotypes from the present *Leptospira* n. sp. (*n* = 11) is in agreement with SNPs observed intraspecifically in other species, like *L. santarosai* and *L. borgpetersenii* (Figure 4). Importantly, haplotypes from the present study are clearly separated from others belonging to an undescribed *Leptospira* species isolated from bovine in the same region (Figure 4).

## 4. Discussion

The present study reinforces the role of bats as reservoirs and dispersers in the dynamics of the transmission cycle of *Leptospira* sp., and sheds light on the knowledge about a still underexplored genetic diversity of leptospires in the Amazon region. We provided, for the first time, genetic sequences from the *sec*Y gene of a potential new pathogenic *Leptospira* n. sp. infecting bats in the region, and, importantly, include new bat hosts species, *Choeroniscus minor* (lesser long-tailed bat) and *Gardnerycteris crenulatum* (striped hairy-nosed bat), for this bacterium. Regarding the remaining hosts identified in the present study, leptospires were previously identified in *Carollia perspicillata* from Colombia, in a cave in the Andes Mountains [28] and from a Caribbean region [29]; in *Artibeus planirostris* and *Myotis riparius* from Peruvian Amazon [16]; in *Artibeus planirostris* from Uraba region, Colombia [30]; in *Uroderma bilobatum* from Caribbean Colombia [29] and Peruvian Amazon [16]; and in *Desmodus rotundus* from Caribbean Colombia [29].

We herein provided genetic evidence based on *rrs* and *sec*Y gene sequences, using different phylogenetic methods and models, for the existence of a new pathogenic species. Although the 16S rRNA gene, the first genetic marker applied for *Leptospira* sp. identification, has been used for a long time as a target for many diagnostic PCR assays [31], it presents a low-taxonomic resolution to differentiate between *Leptospira* species within a clade. This was confirmed in the present study, where no species-specific clusters were observed (Figure 2). Anyway, it was possible to clearly observe the distribution of *Leptospira* sp. haplotypes from bats according to geographic location, bringing important epidemiological inferences (Figure 2). Our sequences from the Amazon region identified in *Uroderma bilobatum*, *Gardnerycteris crenulatum*, and *Desmodus rotundus* clustered together with sequences from *Triaenops menamena* from Madagascar [32] and from Chinese *Myotis* spp. [33]. Further analyses based on markers with better taxonomic resolution are needed to know whether these species in fact constitute a single genetic entity or are distinct. Unfortunately, reference leptospires from bats included in the present study identified by the *rrs* gene do not have associated *sec*Y gene information.

Currently, the *sec*Y gene has been prioritized as a genetic marker for *Leptospira* sp. identification, since it presents a good discriminatory power, and sequence analysis of the gene allows the identification of species, strains, and occasionally genotypes [34]. Interspecific *sec*Y genetic distances between sequences from the present study and the remaining pathogenic species included are equal to or greater than the others previously known (Table 2). Moreover, when including the present sequences within the *L. noguchii* group, the closest species, the intraspecific distance of the clade increases by 150%, reaching a value greater than any *Leptospira* intraspecies distance. Phylogenetic trees and haplotype network corroborated and illustrated clearly the presence of a distinct, highly supported clade, close but separated from *L. noguchii* (Figures 3 and 4). Together, the results suggest genetic evidence for a potentially new species of *Leptospira* circulating in bats from the Brazilian Amazon.

Undoubtedly, a formal naming of the new species involves an integrative approach, including culture and isolation, morphology, serology, and genetic characterization based on multilocus sequence typing (MLST) and whole-genome sequencing (WGS). Unfortunately, leptospires are fastidious and slow-growing organisms, and cultures must be kept and checked for up to 14 weeks [35]. Moreover, we worked in closed forest areas of the Amazon rainforest, and it is not possible to culture the material collected on-site in a sterile environment to optimize culturing. The kidneys were sent frozen to the laboratory, which, added to the fastidious growth of the bacteria, made it unfeasible to cultivate and obtain isolates. Therefore, a complete characterization became a challenge, but efforts will be directed to obtain this formal description.

A high genetic diversity of *Leptospira* sp. circulating in the Amazon region has already been demonstrated based on *sec*Y gene, and the species *L. interrogans*, *L. noguchii*, *L. kirshnerii. L. santarosai*, and *L. borgpetersenii* have been identified in bovine, pigs, rodent, and marsupials [13, 36–39]. Moreover, similarly to the results from the present study, an independent clade, close but separated from *L. kirshnerii* was identified in cattle from the region after phylogenetic analyses based on *sec*Y gene [36].

Leptospirosis is an infectious disease that requires studies on human–animal–environment interface, based on the One Health approach [40]. Evidence for another pathogenic leptospiral species circulating in periurban areas deserves attention, since Brazilian Amazon has been going through intense processes of deforestation, and this generates environmental changes and ecological disturbances that can facilitate human contact with wild species of vectors and reservoirs, favoring the incidence of zoonotic diseases [9]. This becomes even more worrying when the host/carrier is a flying mammal, which can reach long distances, including urban areas, and reinforces the importance of bats as important reservoir hosts and disseminators of multiple pathogenic *Leptospira* sp. [30].

Through a robust genetic analysis, we present evidence for the occurrence of a potentially new species of pathogenic *Leptospira* circulating in the Amazonian biome. Our findings support the high diversity of this genus and contribute to new *Leptospira* taxonomic data about the bat reservoir role that deserves and should be further explored. The characterization of this *Leptospira* n. sp. in bats reinforces the complexity of the transmission dynamics of leptospires, including wild, periurban, and urban environments, emphasizing the need for an integrative look at leptospirosis vigilance within the context of One Health.

## Figures and Tables

**Table 1:** Field, taxonomic and bionomic data about positive bat hosts for *Leptospira* sp. of the present study.

Sample ID	Family	Species	Common name	Locality/municipality	Food guild	Morphology		
						Sex	Forearm (mm)	Wingspan
R21827	Phyllostomidae	*Carollia perspicillata*	Seba's short-tailed bat	Chico Mendes park/Rio Branco	Frugivore	Male	42	33
R21882		*Choeroniscus minor*	Lesser long-tailed bat	Chandless state park/Manoel Urbano	Nectarivore	Male	34	29
R21888		*Artibeus planirostris*	Flat-faced fruiteating bat	Chandless state park/Manoel Urbano	Frugivore	Female	66	48
R21903				Chandless state park/Manoel Urbano	Frugivore	Female	66	49
R21960		*Uroderma bilobatum*	Common tent-making bat	Zoobotanical park/Rio Branco	Frugivore	Male	42.5	44
R21980		*Gardnerycteris crenulatum*	Striped hairy-nosed bat	Piracema forest /Rio Branco	Insectivorous	Female	50	46
R22023		*Desmodus rotundus*	Vampire bat	Roberval Cardoso forest school /Rio Branco	Sanguinivore	Male	55.5	51

R21847	Vespertilionidae	*Myotis riparius*	Riparian myotis	Zoobotanical park/Rio Branco	Insectivore	Male	28	31

**Table 2:** Interspecific genetic distance (bellow diagonal) calculated with the Tamura–Nei parameter between the sequences from this study (represented with  ^*∗*^) and those from GenBank (overall mean between sequences for each species).

Species	*L. alexanderi*	*L. borgpetersenii*	*L. interrogans*	*L. kirshnerii*	*L. noguchii*	*Leptospira* sp. ^*∗*^	*L. weilii*	*L. santarosai*	*L. alstonii*
*L. alexanderi*	*0.01*	0.01	0.03	0.02	0.03	0.03	0.02	0.01	0.02
*L. borgpetersenii*	0.07	*0.01*	0.03	0.03	0.03	0.03	0.02	0.02	0.02
*L. interrogans*	0.21	0.23	*0.00*	0.02	0.02	0.02	0.02	0.03	0.02
*L. kirshnerii*	0.20	0.21	0.11	*0.01*	0.02	0.02	0.02	0.03	0.03
*L. noguchii*	0.21	0.21	0.11	0.11	*0.02*	0.02	0.03	0.03	0.03
*Leptospira* n sp. ^*∗*^	**0.24**	**0.23**	**0.13**	**0.14**	**0.12**	*0.01*	0.03	0.03	0.03
*L. weilii*	0.11	0.12	0.18	0.17	0.21	**0.21**	*0.01*	0.02	0.02
*L. santarosai*	0.10	0.10	0.23	0.20	0.23	**0.25**	0.12	*0.03*	0.02
*L. alstonii*	0.16	0.17	0.17	0.19	0.18	**0.20**	0.19	0.16	*0.00*

Standard deviation is shown above diagonal in underline. The analysis included 428 nucleotide sequences from *sec*Y gene. Genetic distances between sequences from the present study and reference sequences are in bold. Intraspecific genetic distances are in italic along the diagonal.

## Data Availability

The data that support the findings of this study are openly available in GenBank (https://www.ncbi.nlm.nih.gov/genbank/), under acession numbers OQ793707 and OQ793714.

## Supplementary Materials

Supplementary 1 Detailed information about primers used for *Leptospira* sp. identification in the present study.

Supplementary 2 Detailed information of *Leptospira* sp. GenBank sequences from bats included in the present study. Rows in green indicate sequences that clustered together with sequences from the present study. Accession numbers in bold are reference sequences from other hosts.

Supplementary 3 Amino acid alignment of *sec*Y gene from *Leptospira* n. sp. from this study and GenBank sequences from *L. noguchii*. Points indicate identical position with *L. interrogans* strain Fiocruz L1-130 reference sequence. Genetic signatures of *Leptospira* n. sp. are in gray.
