# Supplementary material for: Genetic Evidence for a Potentially New Pathogenic Leptospira sp. Circulating in Bats from Brazilian Amazon
Source: Transbound Emerg Dis. 2023 Sep 19;2023:9677047. doi: 10.1155/2023/9677047 (PMC12016835; doi:10.1155/2023/9677047)
Supplement: Supplementary 1 — Detailed information about primers used for Leptospira sp. identification in the present study. [file 9677047.f1.pdf]

Supplementary Material 1. Detailed information about primers used for *Leptospira* spp. identification in the present study.

Genetic marker	Primer	Sequence (5′–3′)	Reference
*Lip*L32	LipL32-45F<br>LipL32-45R	AAGCATTACCGCTTGTGGTG<br>GAACTCCCATTTCAGCGATT	Stoddard et al., 2009
16S rRNA gene (*rrs*)	Lepto 16S-1st-F<br>Lepto 16S-1st-R<br>Lepto 16S-2nd-F<br>Lepto 16S-2nd-R	CAAGTCAAGCGGAGTAGCAATAC<br>CAACTGGTAGACAACGTTTAGGGC<br>AATCTTCCTYYGAGTCTGGGATAAC<br>TTCACTACCCACGCTTTCGTGCCTC	Ogawa et al., 2015
*sec*Y	secY_outerF<br>secY_outerR<br>secY_inner_F<br>secY_inner_R	ATGCCGATCATTTTTGCTTC<br>CCGTCCCTTAATTTTAGACTTCTTC<br>CCTCAGACGATTATTCAATGGTTATC<br>AGAAGAGAAGTTCCACCGAATG	Grillová et al. 2020
