# Supplementary material for: Genetic Evidence for a Potentially New Pathogenic Leptospira sp. Circulating in Bats from Brazilian Amazon
Source: Transbound Emerg Dis. 2023 Sep 19;2023:9677047. doi: 10.1155/2023/9677047 (PMC12016835; doi:10.1155/2023/9677047)
Supplement: Supplementary 2 — Detailed information of Leptospira sp. GenBank sequences from bats included in the present study. Rows in green indicate sequences that clustered together with sequences from the present study. Accession numbers in bold are reference sequences from other hosts. [file 9677047.f2.pdf]

Supplementary Material 2. Detailed information of *Leptospira* spp. GenBank sequences from bats included in the present study. Rows in green indicate sequences that clustered together with sequences from the present study. Accession numbers in bold are reference sequences from other hosts.

Acession number	Strain/Sample ID	*Leptospira species*	Bat species	Geographical localization	Collection date	Reference
MF498596	SD70	*Leptospira* sp.	*Myotis ricketti*	China	2015	Han et al., 2018
MF498597	SD47	*Leptospira* sp.	*Myotis pequinius*	China	2015	Han et al., 2018
MF498598	SD20	*Leptospira* sp.	*Myotis pequinius*	China	2015	Han et al., 2018
MF498599	SD71	*Leptospira* sp.	*Myotis ricketti*	China	2015	Han et al., 2018
MF498600	SD112	*Leptospira* sp.	*Myotis fimbriatus*	China	2015	Han et al., 2018
MF498601	SD9	*Leptospira* sp.	*Myotis pequinius*	China	2015	Han et al., 2018
MF498602	SD96	*Leptospira* sp.	*Myotis fimbriatus*	China	2015	Han et al., 2018
MF498603	SD92	*Leptospira* sp.	*Myotis ricketti*	China	2015	Han et al., 2018
MF498604	SD7	*Leptospira* sp.	*Myotis pequinius*	China	2015	Han et al., 2018
MF498605	SD88	*Leptospira* sp.	*Myotis fimbriatus*	China	2015	Han et al., 2018
MF498606	SD41	*Leptospira* sp.	*Myotis pequinius*	China	2015	Han et al., 2018
MF498607	SD69	*Leptospira* sp.	*Myotis pequinius*	China	2015	Han et al., 2018
MF498608	SD5	*Leptospira* sp.	*Myotis pequinius*	China	2015	Han et al., 2018
MF498609	SD3	*Leptospira* sp.	*Myotis pequinius*	China	2015	Han et al., 2018
MF498610	SD51	*Leptospira* sp.	*Myotis pequinius*	China	2015	Han et al., 2018
MF498611	SD82	*Leptospira* sp.	*Myotis fimbriatus*	China	2015	Han et al., 2018
MF498612	SD81	*Leptospira* sp.	*Myotis fimbriatus*	China	2015	Han et al., 2018
MF498613	SD84	*Leptospira* sp.	*Myotis fimbriatus*	China	2015	Han et al., 2018
MF498614	SD39	*Leptospira* sp.	*Myotis pequinius*	China	2015	Han et al., 2018
MF498615	SD103	*Leptospira* sp.	*Myotis fimbriatus*	China	2015	Han et al., 2018
MF498616	SD4	*Leptospira* sp.	*Myotis pequinius*	China	2015	Han et al., 2018
MF498617	SD8	*Leptospira* sp.	*Myotis pequinius*	China	2015	Han et al., 2018
MF498618	SD25	*Leptospira* sp.	*Myotis pequinius*	China	2015	Han et al., 2018
MF498619	SD122	*Leptospira* sp.	*Myotis fimbriatus*	China	2015	Han et al., 2018
MF498620	SD80	*Leptospira* sp.	*Myotis fimbriatus*	China	2015	Han et al., 2018
MF498621	SD83	*Leptospira* sp.	*Myotis ricketti*	China	2015	Han et al., 2018
MF498622	SD31	*Leptospira* sp.	*Myotis pequinius*	China	2015	Han et al., 2018
MF498623	SD23	*Leptospira* sp.	*Myotis pequinius*	China	2015	Han et al., 2018
MF498624	SD86	*Leptospira* sp.	*Myotis fimbriatus*	China	2015	Han et al., 2018

MF498625	SD62	*Leptospira* sp.	*Myotis pequinius*	China	2015	Han et al., 2018
MF498626	SD32	*Leptospira* sp.	*Myotis pequinius*	China	2015	Han et al., 2018
MF498627	SD79	*Leptospira* sp.	*Myotis fimbriatus*	China	2015	Han et al., 2018
MF498628	SD21	*Leptospira* sp.	*Myotis pequinius*	China	2015	Han et al., 2018
MF498629	SD102	*Leptospira* sp.	*Myotis fimbriatus*	China	2015	Han et al., 2018
MF498630	SD26	*Leptospira* sp.	*Myotis pequinius*	China	2015	Han et al., 2018
MF498631	SD27	*Leptospira* sp.	*Myotis pequinius*	China	2015	Han et al., 2018
MF498632	SD33	*Leptospira* sp.	*Myotis pequinius*	China	2015	Han et al., 2018
MF498633	SD49	*Leptospira* sp.	*Myotis pequinius*	China	2015	Han et al., 2018
MF498634	SD93	*Leptospira* sp.	*Myotis fimbriatus*	China	2015	Han et al., 2018
MF498635	SD16	*Leptospira* sp.	*Myotis pequinius*	China	2015	Han et al., 2018
MF498636	SD30	*Leptospira* sp.	*Myotis pequinius*	China	2015	Han et al., 2018
MF498637	SD37	*Leptospira* sp.	*Myotis pequinius*	China	2015	Han et al., 2018
MF498638	SD67	*Leptospira* sp.	*Myotis pequinius*	China	2015	Han et al., 2018
MF498639	SD15	*Leptospira* sp.	*Myotis pequinius*	China	2015	Han et al., 2018
MF498640	SD10	*Leptospira* sp.	*Myotis pequinius*	China	2015	Han et al., 2018
MF498641	SD17	*Leptospira* sp.	*Myotis pequinius*	China	2015	Han et al., 2018
MF498642	SD11	*Leptospira* sp.	*Myotis pequinius*	China	2015	Han et al., 2018
MF498643	SD119	*Leptospira* sp.	*Myotis fimbriatus*	China	2015	Han et al., 2018
MF498644	SD90	*Leptospira* sp.	*Myotis ricketti*	China	2015	Han et al., 2018
MF498645	SD73	*Leptospira* sp.	*Myotis pequinius*	China	2015	Han et al., 2018
MF498646	SD38	*Leptospira* sp.	*Myotis pequinius*	China	2015	Han et al., 2018
MF498647	SD40	*Leptospira* sp.	*Myotis pequinius*	China	2015	Han et al., 2018
MF498648	SD89	*Leptospira* sp.	*Myotis fimbriatus*	China	2015	Han et al., 2018
MF498649	SD54	*Leptospira* sp.	*Myotis pequinius*	China	2015	Han et al., 2018
MF498650	SD124	*Leptospira* sp.	*Myotis fimbriatus*	China	2015	Han et al., 2018
MF498651	SD45	*Leptospira* sp.	*Myotis pequinius*	China	2015	Han et al., 2018
MF498652	SD36	*Leptospira* sp.	*Myotis pequinius*	China	2015	Han et al., 2018
MF498653	SD66	*Leptospira* sp.	*Myotis fimbriatus*	China	2015	Han et al., 2018
MF498654	SD63	*Leptospira* sp.	*Myotis ricketti*	China	2015	Han et al., 2018
MF498655	SD44	*Leptospira* sp.	*Myotis pequinius*	China	2015	Han et al., 2018

MF498656	SD85	*Leptospira* sp.	*Myotis fimbriatus*	China	2015	Han et al., 2018
MF498657	SD87	*Leptospira* sp.	*Myotis fimbriatus*	China	2015	Han et al., 2018
KJ607934	10-1D	*L. borgpetersenii*	*Mormopterus francoismoutoui*	Réunion Island	2013	Dietrich et al., 2015
KJ607935	8-16A	*L. borgpetersenii*	*Mormopterus francoismoutoui*	Réunion Island	2013	Dietrich et al., 2015
KJ607936	10-7A	*L. borgpetersenii*	*Mormopterus francoismoutoui*	Réunion Island	2013	Dietrich et al., 2015
KJ607937	1-11e	*L. borgpetersenii*	*Mormopterus francoismoutoui*	Réunion Island	2013	Dietrich et al., 2015
KJ607938	1-9A	*L. borgpetersenii*	*Mormopterus francoismoutoui*	Réunion Island	2013	Dietrich et al., 2015
KJ607939	7-19B	*L. borgpetersenii*	*Mormopterus francoismoutoui*	Réunion Island	2013	Dietrich et al., 2015
KJ607940	3-11C	*L. borgpetersenii*	*Mormopterus francoismoutoui*	Réunion Island	2013	Dietrich et al., 2015
KJ607941	2-16B	*L. borgpetersenii*	*Mormopterus francoismoutoui*	Réunion Island	2013	Dietrich et al., 2015
KJ607942	5-11A	*L. borgpetersenii*	*Mormopterus francoismoutoui*	Réunion Island	2013	Dietrich et al., 2015
KJ607943	10-4B	*L. borgpetersenii*	*Mormopterus francoismoutoui*	Réunion Island	2013	Dietrich et al., 2015
KJ607944	10-3B	*L. borgpetersenii*	*Mormopterus francoismoutoui*	Réunion Island	2013	Dietrich et al., 2015
KJ607945	3-4A	*L. borgpetersenii*	*Mormopterus francoismoutoui*	Réunion Island	2013	Dietrich et al., 2015
AY995713	MMD0955	*L. borgpetersenii*	*Sturnira lilium*	Peru	2003	Matthias et al., 2005
AY995714	MMD1233	*L. borgpetersenii*	*Sturnira tildae*	Peru	2003	Matthias et al., 2005
AY995715	MMD0990	*L. borgpetersenii*	*Desmodus rotundus*	Peru	2003	Matthias et al., 2005
AY995716	MMD1231	*L. borgpetersenii*	*Carollia perspicillata*	Peru	2003	Matthias et al., 2005
AY995717	MMD1220	*Leptospira* sp.	*Artibeus obscurus*	Peru	2003	Matthias et al., 2005
AY995718	MMD1236	*Leptospira* sp.	*Aribeus planirostris*	Peru	2003	Matthias et al., 2005
AY995719	MMD1235	*Leptospira* sp.	*Artibeus planirostris*	Peru	2003	Matthias et al., 2005
AY995720	MMD0965	*Leptospira* sp.	*Rhinophylla pumilio*	Peru	2003	Matthias et al., 2005
AY995721	CPB2568	*Leptospira* sp.	*Glossophaga soricina*	Peru	2002	Matthias et al., 2005
AY995723	MMD1361	*Leptospira* sp.	*Myotis riparius*	Peru	2003	Matthias et al., 2005
AY995724	MMD1239	*Leptospira* sp.	*Lanchophylla thomasi*	Peru	2003	Matthias et al., 2005
AY995725	CPB2650	*L. interrogans*	*Uroderma bilobatum*	Peru	2002	Matthias et al., 2005
AY995726	MMD1388	*L. interrogans*	*Glossophaga soricina*	Peru	2003	Matthias et al., 2005
AY995727	MMD1220	*L. interrogans*	*Artibeus obscurus*	Peru	2003	Matthias et al., 2005
AY995728	MMD1221	*L. interrogans*	*Rhinophylla pumilio*	Peru	2003	Matthias et al., 2005
AY995729	MMD1562	*L. interrogans*	*Promops nasutus*	Peru	2003	Matthias et al., 2005

AY995730	MMD1493	*L. kirschneri*	*Phyllostomus hastatus*	Peru	2003	Matthias et al., 2005
KP822681	580MG	*Leptospira* sp.	*Coleura kibomalandy*	Madagascar	2013	Gomard et al., 2016
KP822682	43MG	*Leptospira* sp.	*Hipposideros commersoni*	Madagascar	2013	Gomard et al., 2016
KP822683	44MG	*Leptospira* sp.	*Hipposideros commersoni*	Madagascar	2013	Gomard et al., 2016
KP822684	45MG	*Leptospira* sp.	*Hipposideros commersoni*	Madagascar	2013	Gomard et al., 2016
KP822685	46MG	*Leptospira* sp.	*Hipposideros commersoni*	Madagascar	2013	Gomard et al., 2016
KP822686	792MG	*Leptospira* sp.	*Hipposideros commersoni*	Madagascar	2013	Gomard et al., 2016
KP822687	794MG	*Leptospira* sp.	*Hipposideros commersoni*	Madagascar	2013	Gomard et al., 2016
KP822689	801MG	*Leptospira* sp.	*Hipposideros commersoni*	Madagascar	2013	Gomard et al., 2016
KP822690	562MG	*Leptospira* sp.	*Hipposideros commersoni*	Madagascar	2013	Gomard et al., 2016
KP822691	48MG	*Leptospira* sp.	*Triaenops menamena*	Madagascar	2013	Gomard et al., 2016
KP822692	95MG	*Leptospira* sp.	*Triaenops menamena*	Madagascar	2013	Gomard et al., 2016
KP822693	733MG	*Leptospira* sp.	*Triaenops menamena*	Madagascar	2013	Gomard et al., 2016
KP822694	133MG	*Leptospira* sp.	*Triaenops menamena*	Madagascar	2013	Gomard et al., 2016
KP822695	134MG	*Leptospira* sp.	*Triaenops menamena*	Madagascar	2013	Gomard et al., 2016
KP822696	138MG	*Leptospira* sp.	*Triaenops menamena*	Madagascar	2013	Gomard et al., 2016
KP822698	157MG	*Leptospira* sp.	*Triaenops menamena*	Madagascar	2013	Gomard et al., 2016
KP822699	161MG	*Leptospira* sp.	*Triaenops menamena*	Madagascar	2013	Gomard et al., 2016
KP822700	176MG	*Leptospira* sp.	*Triaenops menamena*	Madagascar	2013	Gomard et al., 2016
KP822701	177MG	*Leptospira* sp.	*Triaenops menamena*	Madagascar	2013	Gomard et al., 2016
KP822702	178MG	*Leptospira* sp.	*Triaenops menamena*	Madagascar	2013	Gomard et al., 2016
KP822703	184MG	*Leptospira* sp.	*Triaenops menamena*	Madagascar	2013	Gomard et al., 2016
KP822704	185MG	*Leptospira* sp.	*Triaenops menamena*	Madagascar	2013	Gomard et al., 2016
KP822705	461MG	*Leptospira* sp.	*Triaenops menamena*	Madagascar	2013	Gomard et al., 2016
KP822706	483MG	*Leptospira* sp.	*Triaenops menamena*	Madagascar	2013	Gomard et al., 2016
KP822707	566MG	*Leptospira* sp.	*Triaenops menamena*	Madagascar	2013	Gomard et al., 2016
KP822708	567MG	*Leptospira* sp.	*Triaenops menamena*	Madagascar	2013	Gomard et al., 2016
KP822709	568MG	*Leptospira* sp.	*Triaenops menamena*	Madagascar	2013	Gomard et al., 2016
KP822710	49MG	*Leptospira* sp.	*Paratriaenops furculus*	Madagascar	2013	Gomard et al., 2016
KP822711	607MG	*Leptospira* sp.	*Miniopterus sp*	Madagascar	2013	Gomard et al., 2016
KP822712	608MG	*Leptospira* sp.	*Miniopterus sp*	Madagascar	2013	Gomard et al., 2016

KP822713	610MG	*Leptospira* sp.	*Miniopterus sp*	Madagascar	2013	Gomard et al., 2016
KP822714	615MG	*Leptospira* sp.	*Miniopterus sp*	Madagascar	2013	Gomard et al., 2016
KP822715	52MG	*Leptospira* sp.	*Miniopterus sp*	Madagascar	2013	Gomard et al., 2016
KP822716	616MG	*Leptospira* sp.	*Miniopterus sp*	Madagascar	2013	Gomard et al., 2016
KP822717	968MG	*Leptospira* sp.	*Miniopterus sp*	Madagascar	2013	Gomard et al., 2016
KP822718	53MG	*Leptospira* sp.	*Miniopterus sp*	Madagascar	2013	Gomard et al., 2016
KP822719	170MG	*Leptospira* sp.	*Miniopterus griffithsi*	Madagascar	2013	Gomard et al., 2016
KP822720	174MG	*Leptospira* sp.	*Miniopterus griffithsi*	Madagascar	2013	Gomard et al., 2016
KP822721	183MG	*Leptospira* sp.	*Miniopterus griffithsi*	Madagascar	2013	Gomard et al., 2016
KP822722	36MG	*Leptospira* sp.	*Miniopterus mahafaliensis*	Madagascar	2013	Gomard et al., 2016
KP822723	76MG	*Leptospira* sp.	*Miniopterus mahafaliensis*	Madagascar	2013	Gomard et al., 2016
KP822724	99MG	*Leptospira* sp.	*Miniopterus mahafaliensis*	Madagascar	2013	Gomard et al., 2016
KP822725	118MG	*Leptospira* sp.	*Miniopterus mahafaliensis*	Madagascar	2013	Gomard et al., 2016
KP822726	50MG	*Leptospira* sp.	*Miniopterus mahafaliensis*	Madagascar	2013	Gomard et al., 2016
KP822727	120MG	*Leptospira* sp.	*Miniopterus mahafaliensis*	Madagascar	2013	Gomard et al., 2016
KP822728	122MG	*Leptospira* sp.	*Miniopterus mahafaliensis*	Madagascar	2013	Gomard et al., 2016
KP822729	123MG	*Leptospira* sp.	*Miniopterus mahafaliensis*	Madagascar	2013	Gomard et al., 2016
KP822730	741MG	*Leptospira* sp.	*Miniopterus mahafaliensis*	Madagascar	2013	Gomard et al., 2016
KP822731	743MG	*Leptospira* sp.	*Miniopterus mahafaliensis*	Madagascar	2013	Gomard et al., 2016
KP822732	749MG	*Leptospira* sp.	*Miniopterus mahafaliensis*	Madagascar	2013	Gomard et al., 2016
KP822733	750MG	*Leptospira* sp.	*Miniopterus mahafaliensis*	Madagascar	2013	Gomard et al., 2016
KP822734	753MG	*Leptospira* sp.	*Miniopterus mahafaliensis*	Madagascar	2013	Gomard et al., 2016
KP822735	950MG	*Leptospira* sp.	*Miniopterus mahafaliensis*	Madagascar	2013	Gomard et al., 2016
KP822736	755MG	*Leptospira* sp.	*Miniopterus mahafaliensis*	Madagascar	2013	Gomard et al., 2016
KP822737	757MG	*Leptospira* sp.	*Miniopterus mahafaliensis*	Madagascar	2013	Gomard et al., 2016
KP822738	796MG	*Leptospira* sp.	*Miniopterus mahafaliensis*	Madagascar	2013	Gomard et al., 2016
KP822739	142MG	*Leptospira* sp.	*Miniopterus mahafaliensis*	Madagascar	2013	Gomard et al., 2016
KP822740	145MG	*Leptospira* sp.	*Miniopterus mahafaliensis*	Madagascar	2013	Gomard et al., 2016
KP822741	146MG	*Leptospira* sp.	*Miniopterus mahafaliensis*	Madagascar	2013	Gomard et al., 2016
KP822742	150MG	*Leptospira* sp.	*Miniopterus mahafaliensis*	Madagascar	2013	Gomard et al., 2016
KP822743	151MG	*Leptospira* sp.	*Miniopterus mahafaliensis*	Madagascar	2013	Gomard et al., 2016

KP822744	152MG	*Leptospira* sp.	*Miniopterus mahafaliensis*	Madagascar	2013	Gomard et al., 2016
KP822745	188MG	*Leptospira* sp.	*Miniopterus mahafaliensis*	Madagascar	2013	Gomard et al., 2016
KP822746	191MG	*Leptospira* sp.	*Miniopterus mahafaliensis*	Madagascar	2013	Gomard et al., 2016
KP822747	897MG	*Leptospira* sp.	*Miniopterus mahafaliensis*	Madagascar	2013	Gomard et al., 2016
KP822748	728MG	*Leptospira* sp.	*Miniopterus majori*	Madagascar	2013	Gomard et al., 2016
KP822749	115MG	*Leptospira* sp.	*Miniopterus sororculus*	Madagascar	2013	Gomard et al., 2016
KP822750	724MG	*Leptospira* sp.	*Miniopterus sororculus*	Madagascar	2013	Gomard et al., 2016
KP822751	736MG	*Leptospira* sp.	*Miniopterus sororculus*	Madagascar	2013	Gomard et al., 2016
KP822752	772MG	*Leptospira* sp.	*Miniopterus sororculus*	Madagascar	2016	Gomard et al., 2016
KP822753	773MG	*Leptospira* sp.	*Miniopterus sororculus*	Madagascar	2016	Gomard et al., 2016
KP822754	783MG	*Leptospira* sp.	*Miniopterus sororculus*	Madagascar	2016	Gomard et al., 2016
KP822755	785MG	*Leptospira* sp.	*Miniopterus sororculus*	Madagascar	2016	Gomard et al., 2016
KP822756	790MG	*Leptospira* sp.	*Miniopterus sororculus*	Madagascar	2016	Gomard et al., 2016
KP822757	92MG	*Leptospira* sp.	*Chaerephon leucogaster*	Madagascar	2016	Gomard et al., 2016
KP822758	8MG	*Leptospira* sp.	*Mormopterus jugularis*	Madagascar	2016	Gomard et al., 2016
KP822759	12MG	*Leptospira* sp.	*Mormopterus jugularis*	Madagascar	2016	Gomard et al., 2016
KP822760	13MG	*Leptospira* sp.	*Mormopterus jugularis*	Madagascar	2016	Gomard et al., 2016
KP822761	15MG	*Leptospira* sp.	*Mormopterus jugularis*	Madagascar	2016	Gomard et al., 2016
KP822762	31MG	*Leptospira* sp.	*Mormopterus jugularis*	Madagascar	2016	Gomard et al., 2016
KP822763	20MG	*Leptospira* sp.	*Mormopterus jugularis*	Madagascar	2016	Gomard et al., 2016
KP822764	108MG	*Leptospira* sp.	*Mormopterus jugularis*	Madagascar	2016	Gomard et al., 2016
KP822765	110MG	*Leptospira* sp.	*Mormopterus jugularis*	Madagascar	2016	Gomard et al., 2016
KP822766	762MG	*Leptospira* sp.	*Mormopterus jugularis*	Madagascar	2016	Gomard et al., 2016
KP822767	165MG	*Leptospira* sp.	*Mormopterus jugularis*	Madagascar	2016	Gomard et al., 2016
KP822768	172MG	*Leptospira* sp.	*Mormopterus jugularis*	Madagascar	2016	Gomard et al., 2016
KP822769	204MG	*Leptospira* sp.	*Mormopterus jugularis*	Madagascar	2016	Gomard et al., 2016
KP822770	206MG	*Leptospira* sp.	*Mormopterus jugularis*	Madagascar	2016	Gomard et al., 2016
KP822771	209MG	*Leptospira* sp.	*Mormopterus jugularis*	Madagascar	2016	Gomard et al., 2016
KP822772	911MG	*Leptospira* sp.	*Mormopterus jugularis*	Madagascar	2016	Gomard et al., 2016
KP822773	68MG	*Leptospira* sp.	*Otomops madagascariensis*	Madagascar	2016	Gomard et al., 2016
KP822774	71MG	*Leptospira* sp.	*Otomops madagascariensis*	Madagascar	2016	Gomard et al., 2016

KP822775	418MG	*Leptospira* sp.	*Otomops madagascariensis*	Madagascar	2016	Gomard et al., 2016
KP822776	407MG	*Leptospira* sp.	*Rousettus madagascariensis*	Madagascar	2016	Gomard et al., 2016
KP822777	453MG	*Leptospira* sp.	*Rousettus madagascariensis*	Madagascar	2016	Gomard et al., 2016
KP822778	459MG	*Leptospira* sp.	*Rousettus madagascariensis*	Madagascar	2016	Gomard et al., 2016
KP822779	525MG	*Leptospira* sp.	*Rousettus madagascariensis*	Madagascar	2016	Gomard et al., 2016
KP822780	530MG	*Leptospira* sp.	*Rousettus madagascariensis*	Madagascar	2016	Gomard et al., 2016
KP822781	531MG	*Leptospira* sp.	*Rousettus madagascariensis*	Madagascar	2016	Gomard et al., 2016
KP822782	532MG	*Leptospira* sp.	*Rousettus madagascariensis*	Madagascar	2016	Gomard et al., 2016
KP822783	632MG	*Leptospira* sp.	*Rousettus madagascariensis*	Madagascar	2016	Gomard et al., 2016
KP822784	718MG	*Leptospira* sp.	*Myotis goudoti*	Madagascar	2016	Gomard et al., 2016
KP822785	721MG	*Leptospira* sp.	*Myotis goudoti*	Madagascar	2016	Gomard et al., 2016
KP822786	739MG	*Leptospira* sp.	*Myotis goudoti*	Madagascar	2016	Gomard et al., 2016
KP822787	490MG	*Leptospira* sp.	*Myotis goudoti*	Madagascar	2016	Gomard et al., 2016
KP822788	491MG	*Leptospira sp.*	*Myotis goudoti*	Madagascar	2016	Gomard et al., 2016
KP822789	493MG	*Leptospira* sp.	*Myotis goudoti*	Madagascar	2016	Gomard et al., 2016
KP822790	494MG	*Leptospira* sp.	*Myotis goudoti*	Madagascar	2016	Gomard et al., 2016
KP822791	495MG	*Leptospira* sp.	*Myotis goudoti*	Madagascar	2016	Gomard et al., 2016
KP822792	558MG	*Leptospira* sp.	*Myotis goudoti*	Madagascar	2016	Gomard et al., 2016
KP822793	594MG	*Leptospira* sp.	*Myotis goudoti*	Madagascar	2016	Gomard et al., 2016
KP822794	55MG	*Leptospira* sp.	*Myotis goudoti*	Madagascar	2016	Gomard et al., 2016
KP822795	857MG	*Leptospira* sp.	*Neoromicia robertsi*	Madagascar	2016	Gomard et al., 2016
KP822796	464MG	*Leptospira* sp.	*Scotophilus marovaza*	Madagascar	2016	Gomard et al., 2016
JQ288729	C54	*Leptospira* sp.	*Otomops madagascariensis*	Madagascar	2012	Lagadec et al., 2012
JQ288730	Lep1	*Leptospira* sp.	*Miniopterus mahafaliensis*	Madagascar	2012	Lagadec et al., 2012
JQ288731	Lep2	*Leptospira* sp.	*Triaenops menamena*	Madagascar	2012	Lagadec et al., 2012
JQ288732	Lep3	*Leptospira* sp.	*Rousettus obliviosus*	Comoros	2012	Lagadec et al., 2012
JQ288733	V50	*Leptospira* sp.	*Rousettus obliviosus*	Comoros	2012	Lagadec et al., 2012
JQ288734	Lep4	*Leptospira* sp.	*Miniopterus griveaudi*	Comoros	2012	Lagadec et al., 2012
LC005173	ZFB08-09	*Leptospira sp.*	*Eidolon helvum*	Zambia	2008	Ogawa et al., 2015
LC005174	ZFB08-21	*Leptospira* sp.	*Eidolon helvum*	Zambia	2008	Ogawa et al., 2015
LC005175	ZFB08-40	*Leptospira* sp.	*Eidolon helvum*	Zambia	2008	Ogawa et al., 2015

LC005176	ZFB08-44	*Leptospira* sp.	*Eidolon helvum*	Zambia		2008	Ogawa et al., 2015
LC005177	ZFB08-48	*Leptospira* sp.	*Eidolon helvum*	Zambia		2008	Ogawa et al., 2015
LC005178	ZFB08-50	*Leptospira* sp.	*Eidolon helvum*	Zambia		2008	Ogawa et al., 2015
LC005179	ZFB08-62	*Leptospira* sp.	*Eidolon helvum*	Zambia		2008	Ogawa et al., 2015
LC005180	ZFB08-79	*Leptospira* sp.	*Eidolon helvum*	Zambia		2008	Ogawa et al., 2015
LC005181	ZFB08-91	*Leptospira* sp.	*Eidolon helvum*	Zambia		2008	Ogawa et al., 2015
LC005182	ZFB08-92	*Leptospira* sp.	*Eidolon helvum*	Zambia		2008	Ogawa et al., 2015
LC005183	ZFB08-95	*Leptospira* sp.	*Eidolon helvum*	Zambia		2008	Ogawa et al., 2015
LC005184	ZFB08-96	*Leptospira* sp.	*Eidolon helvum*	Zambia		2008	Ogawa et al., 2015
LC005185	ZFB09-04	*Leptospira* sp.	*Eidolon helvum*	Zambia		2009	Ogawa et al., 2015
LC005186	ZFB09-25	*Leptospira* sp.	*Eidolon helvum*	Zambia		2009	Ogawa et al., 2015
LC005187	ZFB10-28	*Leptospira* sp.	*Eidolon helvum*	Zambia		2010	Ogawa et al., 2015
LC005188	ZFB10-36	*Leptospira* sp.	*Eidolon helvum*	Zambia		2010	Ogawa et al., 2015
LC005189	ZFB10-49	*Leptospira* sp.	*Eidolon helvum*	Zambia		2010	Ogawa et al., 2015
LC005190	ZFB11-95	*Leptospira* sp.	*Eidolon helvum*	Zambia		2011	Ogawa et al., 2015
LC005191	ZFB12-56	*Leptospira* sp.	*Eidolon helvum*	Zambia		2012	Ogawa et al., 2015
LC005192	ZFB12-78	*Leptospira* sp.	*Eidolon helvum*	Zambia		2012	Ogawa et al., 2015
LC005193	ZFB12-96	*Leptospira* sp.	*Eidolon helvum*	Zambia		2012	Ogawa et al., 2015
LC005194	ZFB12-103	*Leptospira* sp.	*Eidolon helvum*	Zambia		2012	Ogawa et al., 2015
LC005195	ZFB12-105	*Leptospira* sp.	*Eidolon helvum*	Zambia		2012	Ogawa et al., 2015
LC005196	ZFB13-102	*Leptospira* sp.	*Eidolon helvum*	Zambia		2013	Ogawa et al., 2015
LC005197	ZFB13-104	*Leptospira* sp.	*Eidolon helvum*	Zambia		2013	Ogawa et al., 2015
LC005198	ZFB13-106	*Leptospira* sp.	*Eidolon helvum*	Zambia		2013	Ogawa et al., 2015
LC005199	ZFB13-107	*Leptospira* sp.	*Eidolon helvum*	Zambia		2013	Ogawa et al., 2015
Present study	R21960	*Leptospira* sp.	*Uroderma bilobatum*	Brazil		2021	Present study
Present study	R21980	*Leptospira* sp.	*Striped hairy-nosed bat*	Brazil		2021	Present study
Present study	R22023	*Leptospira* sp.	*Desmodus rotundus*	Brazil		2021	Present study
**DQ483058**	94-79970/3	*L. weilli*	Bovine	Australia	NP		Slack et al., 2008
**KJ847187**	200901116	*L. mayottensis*	Human	France	NP		Picareau et al., 2014
**KC662453**	KB1105K	*L. borgpetersenii*	Rodent	Thailand		2011	Krairojananan et al., 2013
**JQ988836**	Manhao 3_DB53	*L. alexanderi*	NP	Germany	NP		Rettinger et al., 2012

**EF536998**	H2	*L. kirschneri*	Human	Thailand	NP	Thaipadungpanit et al., 2007
**CP012603**	Norma	*L. interrogans*	Bovine	Brazil	NP	Cosate et al., 2015
**CP006694**	LT 821	*L. santarosai*	NP	NP	NP	Chou et al., 2014
**FJ154587**	LSU 1945 16S	*L. noguchii*	Human	Brazil	NP	Cerqueira et al., 2010

NP: Not provided
