# Supplementary material for: Genetic Evidence for a Potentially New Pathogenic Leptospira sp. Circulating in Bats from Brazilian Amazon
Source: Transbound Emerg Dis. 2023 Sep 19;2023:9677047. doi: 10.1155/2023/9677047 (PMC12016835; doi:10.1155/2023/9677047)
Supplement: Supplementary 3 — Amino acid alignment of secY gene from Leptospira n. sp. from this study and GenBank sequences from L. noguchii. Points indicate identical position with L. interrogans strain Fiocruz L1-130 reference sequence. Genetic signatures of Leptospira n. sp. are in gray. [file 9677047.f3.pdf]

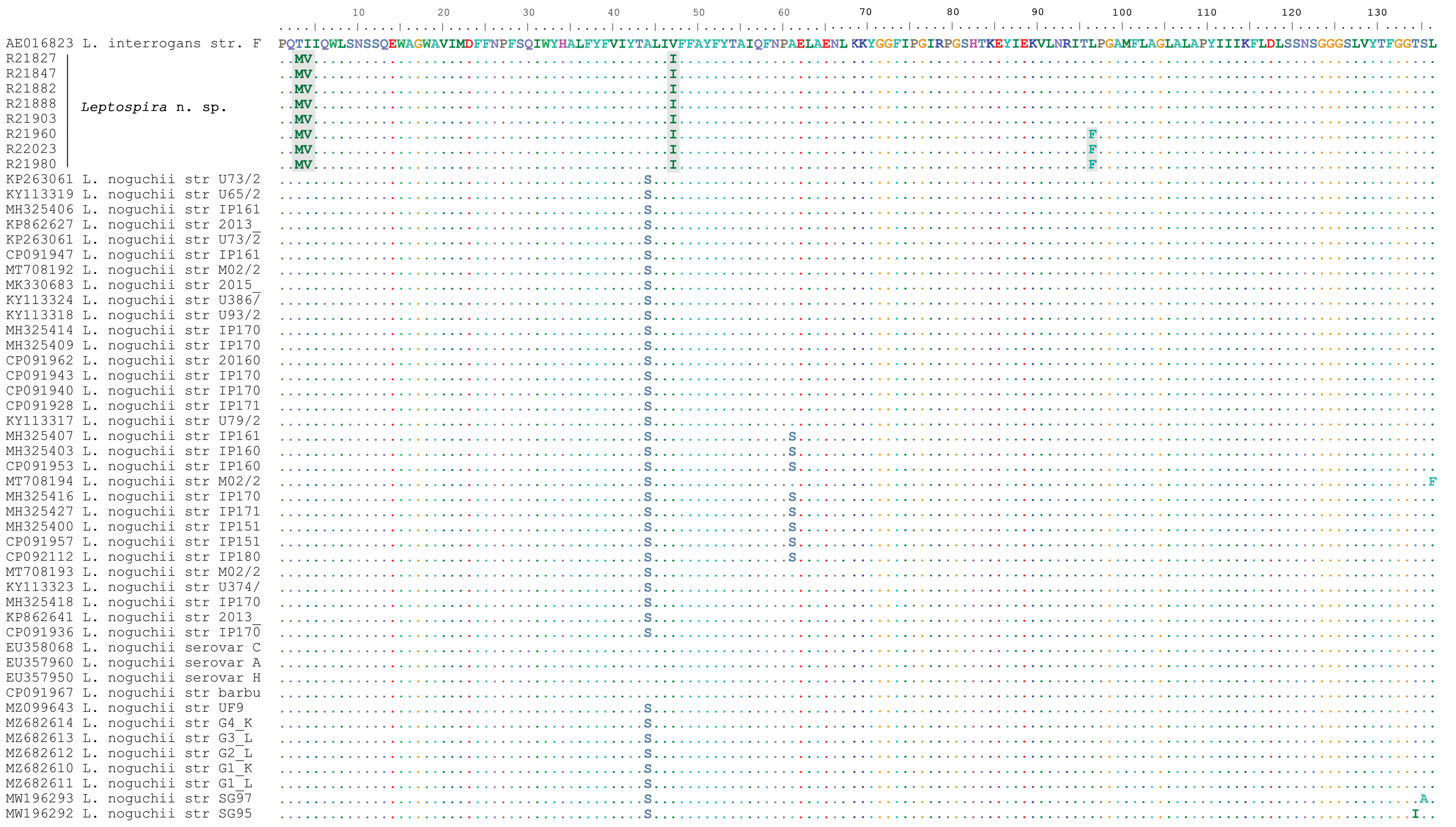

AE016823 L. interrogans str. F
PQTIIQWLSNSSQEWAGWAVIMDFFNPFSQIWYHALFYFVIYTALIVFFAYFYTAIQFNPAELAENL KKYGGFIPGIRPGSHTKEYIEKVLNRITLPGAMFLAGLALAPYIIIKFLDLSSNSGGGSLVYTFGGTSL
R21827
R21847
R21882
R21888
R21903
R21960
R22023
R21980
Leptospira n. sp.
KP263061 L. noguchii str U73/2
KY113319 L. noguchii str U65/2
MH325406 L. noguchii str IP161
KP862627 L. noguchii str 2013_
KP263061 L. noguchii str U73/2
CP091947 L. noguchii str IP161
MT708192 L. noguchii str M02/2
MK330683 L. noguchii str 2015_
KY113324 L. noguchii str U386/
KY113318 L. noguchii str U93/2
MH325414 L. noguchii str IP170
MH325409 L. noguchii str IP170
CP091962 L. noguchii str 20160
CP091943 L. noguchii str IP170
CP091940 L. noguchii str IP170
CP091928 L. noguchii str IP171
KY113317 L. noguchii str U79/2
MH325407 L. noguchii str IP161
MH325403 L. noguchii str IP160
CP091953 L. noguchii str IP160
MT708194 L. noguchii str M02/2
MH325416 L. noguchii str IP170
MH325427 L. noguchii str IP171
MH325400 L. noguchii str IP151
CP091957 L. noguchii str IP151
CP092112 L. noguchii str IP180
MT708193 L. noguchii str M02/2
KY113323 L. noguchii str U374/
MH325418 L. noguchii str IP170
KP862641 L. noguchii str 2013_
CP091936 L. noguchii str IP170
EU358068 L. noguchii serovar C
EU357960 L. noguchii serovar A
EU357950 L. noguchii serovar H
CP091967 L. noguchii str barbu
MZ099643 L. noguchii str UF9
MZ682614 L. noguchii str G4_K
MZ682613 L. noguchii str G3_L
MZ682612 L. noguchii str G2_L
MZ682610 L. noguchii str G1_K
MZ682611 L. noguchii str G1_L
MW196293 L. noguchii str SG97
MW196292 L. noguchii str SG95
